# Fourier transform infrared microspectroscopy monitoring of 5-fluorouracil-induced apoptosis in SW620 colon cancer cells

**DOI:** 10.3892/mmr.2014.3088

**Published:** 2014-12-12

**Authors:** YANFENG GAO, XIONGWEI HUO, LIU DONG, XUEJUN SUN, HE SAI, GUANGBING WEI, YIZHUANG XU, YUANFU ZHANG, JINGUANG WU

**Affiliations:** 1 Department of Anesthesiology, The First Affiliated Hospital of Medical College, Xi’an Jiaotong University, Xi’an, Shaanxi 710061, P.R. China; 2 Department of General Surgery, The First Affiliated Hospital of Medical College, Xi’an Jiaotong University, Xi’an, Shaanxi 710061, P.R. China; 3 Department of General Surgery, Shaanxi Provincial People’s Hospital, Xi’an, Shaanxi 710068, P.R. China; 4 Beijing National Laboratory for Molecular Sciences, State Key Laboratory for Rare Earth Materials Chemistry and Applications, College of Chemistry and Molecular Engineering, Peking University, Beijing 100871, P.R. China

**Keywords:** colon cancer, cell cycle, apoptosis, Fourier transform infrared, chemotherapy

## Abstract

Colon cancer is associated with a high incidence and a poor prognosis. The aim of the present study was to determine whether Fourier transform infrared (FTIR) microspectroscopy can be used to monitor the chemotherapy drug-induced apoptosis of SW620 colon cancer cells. The 50% inhibitory concentration (IC_50_) of 5-fluorouracil (5-FU), the main chemotherapeutic agent used for the treatment of colorectal cancer, was determined as the inhibition of growth of the SW620 cells using an MTT assay. Cell starvation and 5-FU treatment synergized to arrest the cells in the G1 and S phases of the cell cycle. FTIR combined with fluorescence activated cell sorting (FACS) analysis were used to analyze the SW620 cells following treatment with 5-FU for 12, 24 and 48 h. The apoptotic cells had several spectral characteristics. The relative peak intensity ratio (*I*_1740_/*I*_1460_) was significantly increased (P<0.05), the I_1740_/I_1460_ ratio, associated with a band of amino acid residues at 1,410 cm^−1^ was significantly increased at the early and late phases of cell death (P<0.05), the peaks at 1,240 cm^−1^ increased in wave number, a band at 1,040 cm^−1^, associated with polysaccharides, appeared at 24 and 48 h and then moved to a higher wave number and the *I*_1040_/*I*_1460_ ratio increased at the late stage of apoptosis. These results demonstrated that FTIR can be used as a label-free technique to monitor cancer cell apoptosis and to understand the spectral fingerprints of apoptotic cells. This suggested that FTIR spectral features have potential as a powerful tool to monitor cancer cell apoptosis.

## Introduction

Cancer remains one of the major life threatening diseases in humans. Each year, almost 70,000 individuals between the ages of 15 and 40 years are diagnosed with cancer in the USA (1). Amongst all types of cancer, colorectal cancer is the second most frequent cause of cancer-associated mortality in developed countries and is the fifth leading cause of mortality in China (2,3). Surgery and chemotherapy are the main techniques used in the treatment of colorectal cancer and 5-fluorouracil (5-FU) is the chemotherapeutic agent of choice for its treatment (4). It has been widely used for the treatment of solid tumors, including colorectal, breast and head and neck cancer (5). On entering the tumor cell, 5-FU can exert cytotoxic effects via the inhibition of thymidylate synthetase (TS) or through incorporation into RNA and DNA, which lead to the activation of apoptosis (6,7). Apoptosis occurs as part of various normal and pathological processes and can be triggered by chemotherapeutic drugs (8). Apoptosis is the process by which cells self-destruct without evoking an inflammatory response and is characterized by nuclear and cytoplasmic shrinkage, chromatin condensation, internucleosomal DNA cleavage and plasma membrane blebbing (9,10).

At present, colorectal cancer is monitored following chemotherapy using techniques, including electronic colon endoscopy, computer-assisted tomography and B ultrasonic imaging. The disadvantage of these approaches is of that auxiliary examination can only monitor the level of morphological changes, but not molecular changes, including gene mutations. Although molecular biological techniques focusing on the molecular mechanism of colorectal cancer have improved, there are few specific biomarkers and these techniques are time-consuming (11). Thus, it is important to develop an accurate, simple, rapid and non-invasive technique for the real-time monitoring of colon cancer chemotherapy at the molecular level.

Following advances in vibrational spectroscopy techniques, their application in medical biology is increasing (12). Fourier transform infrared spectroscopy (FTIR) has been used as an analytical tool in chemistry for several years. In addition, FTIR can be applied as a simple, rapid and non-invasive method to detect and identify cancer with minimal sample preparation and can be used for the qualitative identification and quantitative analysis of various components in a complex mixture (13). Few studies have monitored the morphology of cancer cells using vibrational spectroscopy (14–17) and, although several spectroscopic studies have examined the cell apoptosis, cell cycle, differentiation and proliferation of different cell lines and tissues (18,19), there has been no detailed analysis of colon cancer cell apoptosis following treatment with chemotherapeutic agents. In the present study, the feasibility of FTIR spectroscopy analysis to identify different stages of tumor cells apoptosis was assessed to attempt to provide a theoretical basis for the application of FTIR spectroscopy in the monitoring of clinical chemotherapy.

## Materials and methods

### Cell lines and cell culture

The human colon carcinoma cell line SW620, also termed CCL_227_, which overexpresses TS, was purchased from the China Center for Type Culture Collection, (Wuhan, China) (20). The cells were grown in high glucose RPMI medium (Hyclone Laboratories, Inc., Logan, UT, USA) supplemented with 10% fetal calf serum (Gibco-BRL, Carlsbad, CA, USA) with penicillin and streptomycin (100 U/ml each; Sigma-Aldrich, St. Louis, MO, USA) in a humidified incubator with 5% CO_2_ at 37°C. All experiments were performed on exponentially growing cells. All cells were incubated with 5-FU (Sigma-Aldrich) for 12, 24 and 48 h to induce apoptosis.

### 5-FU treatment

The cells in 100 μl culture medium per well were seeded into 96-well plates (Gibco-BRL) and cultured at 37°C for 24 h. The culture medium was then replaced with serum-free medium and various concentrations of 5-FU (6.25, 12.5, 25, 50, 100, 200 and 400 μM) were adjusted in the wells. Following additional incubation at 37°C for 24 h, 10 μl MTT (Sigma-Aldrich) dissolved in phosphate-buffered saline (PBS) at a concentration of 5 mg/ml was added to each well and the plates were incubated at 37°C for 4 h. The medium was removed and 150 μl dimethylsulfoxide (Sigma-Aldrich) was added to each well followed by agitation of the plates for 5 min. The absorbance was then measured at 570 nm in a scanning spectrophotometer (Spectronic 20D; Milton Roy, Rochester, NY, USA). A total of six wells were used for each drug concentration and the experiment was repeated three times. The 50% inhibitory concentration (IC_50_) was then calculated from the survival curves using GraphPad Prism 5 (San Diego, CA, USA).

### Cell cycle analysis by FACS

The tumor cells were seeded (2×10^5^ cells/well) into six-well plates. Following overnight incubation, the medium was replaced with serum-free medium containing 50 μM 5-FU for 24 h for serum starvation and incubated for 12, 24 or 48 h. Starving the cells of serum prior to treatment arrests them at the G1/S phase of the cell cycle for synchronization in order to minimize spectral differences at 1,080 cm^−1^, which can be attributed to cells being in different phases of the cell cycle (21–23). The cells were then harvested and stained with propidium iodide (PI) for cell cycle and apoptotic analysis using standard FACS techniques with a FACSort flow cytometer (BD Biosciences, Franklin Lakes, NJ, USA) according to the manufacturer’s instructions. The results were analyzed and expressed as percentages of the total gated cells using Modifit LT™ software (BD Biosciences).

### Isolation of nuclei

The SW620 cells were harvested by scraping the plates in cold PBS and were collected by centrifugation at 666 × g. The cells were then resuspended in three volumes with respect to the cell pellet of the hypotonic buffer CelLytic^TM^ NuCLEAR^TM^ Extraction kit (Sigma, USA) (24). Following incubation on ice for 15 min, 5% Triton X-100 was added and a cytosolic fraction was prepared by centrifuging the sample for 30 sec at 4°C at 1,498 × g and subsequently clarified by centrifugation at 666 × g for 15 min at 4°C. The nuclear pellets were washed twice in hypotonic buffer to remove any residual cytosolic contamination and then resuspended in one volume of nuclear lysis buffer. The cells were then incubated for 30 min at 4°C with gentle agitation and finally centrifuged for 30 min at 4°C at 1,498 × g.

### FTIR microspectroscopy (MSP) collection

FTIR-MSP is similar to visible light microscopy; however, it does not use glass refractive elements (glass is opaque to infrared light of λ>5 μm) (12). For this reason, microtransmission FTIR requires samples to be deposited on optical windows, such as CaF_2_, which do not absorb infared light (25,26). The FTIR spectrometer was equipped with a liquid nitrogen-cooled mercury cadmium telluride (MCT) detector and a KBr beam splitter (Thermo Nicolet 6700; Thermo Fisher Scientific, Waltham, MA, USA). All cells were grown on a CaF_2_ window film. A blank BaF_2_ window-film was scanned as background and the sample was then scanned. To collect the data for each spectrum, 32 scans were performed in the mid-infrared range (wave number, 4,000-900 cm^−1^) at a resolution of 8 cm^−1^.

### Statistical analysis

OMNIC 8.0 (Thermo Fisher Scientific) was used as data-processing software for FTIR spectrum analysis (27). The five point moving average smoothing method was used for each spectrum to reduce random noise. The intensity and peak position of each band was measured using Spapro version 2.2 software (College of Chemistry and Molecular Engineering, Peking University, Beijing, China). All experiments were repeated at least three times. The statistical significance of the differences were evaluated by one way analysis of variance and P<0.05 was considered to indicate a statistically significant difference.

## Results

### Concentration of 5-FU

**Figure 1:**
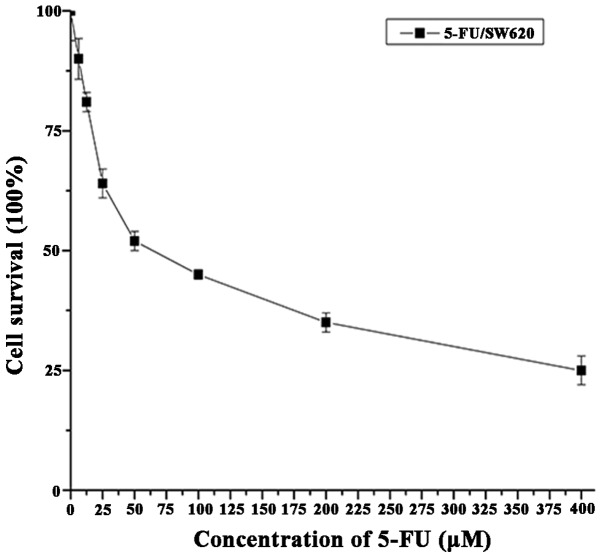
Proliferative activity of the 5-FU-treated SW620 cells at 5-FU concentrations of 6.25, 12.5, 25, 50, 100, 200 and 400 μM, assessed after 24 h using an MTT assay. The y-axis represents the cell survival, calculated as the ratio between the 5-FU-treated and control cells. 5-FU, 5-fluorouracil.

Following treatment of the SW620 cells with increasing concentrations of 5-FU, cell growth was inhibited to different degrees and concentration-dependent cell toxicity was observed (Fig. 1). The IC_50_ data indicated the 5-FU-resistance levels of the SW-620 cells at IC_50_ 50 μM (Fig. 1). Therefore, 50 μM 5-FU was selected as the concentration for use in the subsequent experiments.

### Sw620 cells arrest at the G1 and S phase

**Figure 2:**
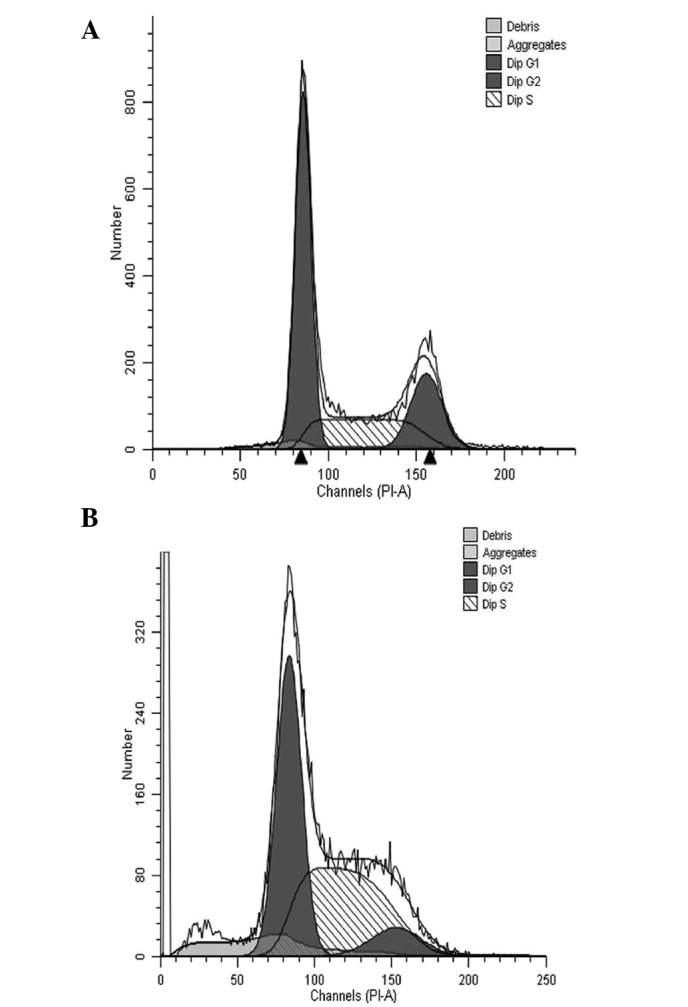
Cell cycle arrest at the G1/S phase was evaluated by fluorescence activated cell sorting using Annexin V/PI staining in the SW620 cells in the (A) control and (B) following serum-starving. PI, propidium iodide.

The SW620 cells were serum-starved and analyzed by flow cytometry following PI staining, which resulted in S and G1 phase cell cycle arrest with 90% cells in the G1+S phases as compared with 77% in the control (Fig. 2). Compared with the control, S and G1 phase arrest in the serum-starved SW-620 cells was significantly increased (P<0.05).

### Apoptosis induced by 5-FU

**Figure 3:**
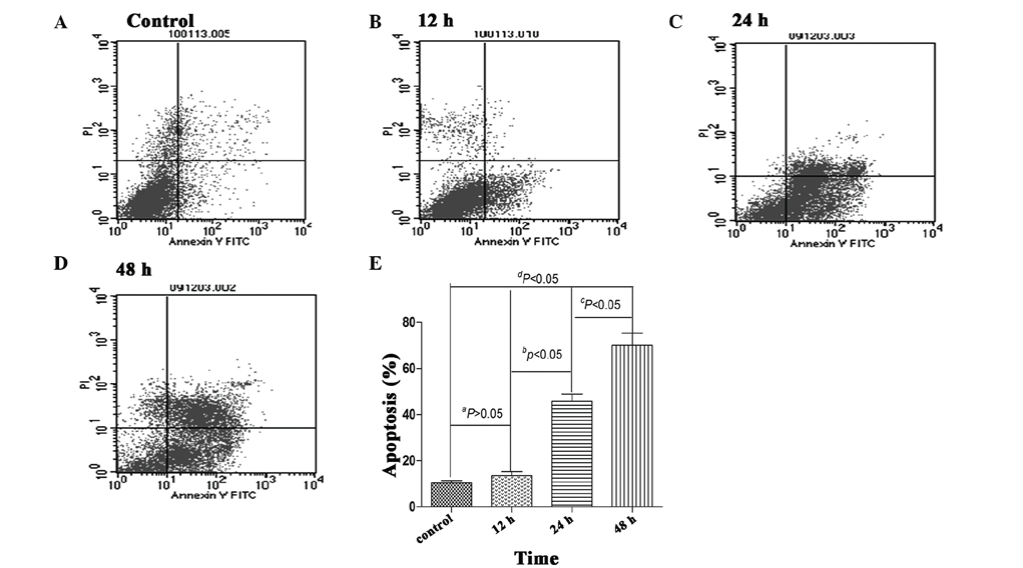
Apoptosis in the (A) control SW620 cells and in SW620 cells induced by 5-FU (50 μM) for (B) 12 h (C) 24 h and (D) 48 h compared with the control. (E) Quantification of apoptosis in the SW620 cells. ^a^12 h, vs. control; ^b^24 h, vs. 12 h; ^c^48 h, vs. 24 h; ^d^48 and 24 h vs. 12 h and control. 5-FU, 5-fluorouracil.

Treatment with 5-FU induced SW620 cell apoptosis. The population of apoptotic cells was evaluated by FACS using Annexin V/PI staining following 5-FU treatment at 50 μM for 12, 24 and 48 h. Annexin V is a Ca^2+^-dependent phospholipid binding protein, which can combine with the apoptotic cell valgus membrane, and PI is a nucleic acid-binding dye. PI can distinguish between early and late stage apoptosis using FACS. The percentage of Annexin V-positive apoptotic cells increased following 5-FU treatment compared with that of the control (10.04±2.1%; Fig. 3A), for 12 h (Fig. 3B; 13.74±3.2%), 24 h (Fig. 3C; 64.77±7.5%) and 48 h (Fig. 3D; 70.41±9.2%). As shown in Fig. 3E, a significant increase in apoptosis was observed between the control and 12 h, 12 and 24 h (early apoptosis) and between 24 and 48 h (late apoptosis).

### FTIR-MSP spectral analysis

**Figure 4:**
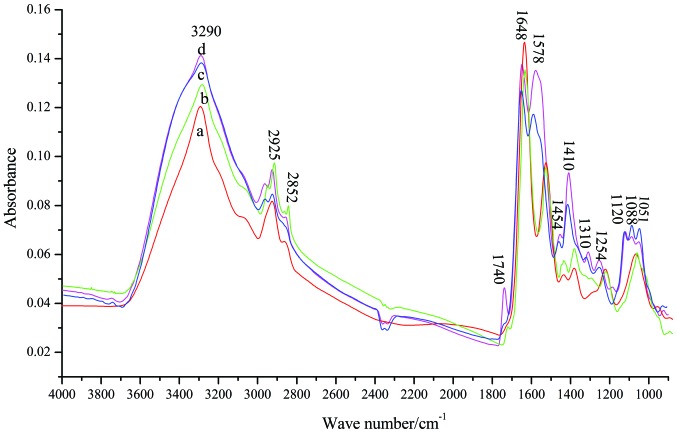
Fourier transform infrared spectra at wave numbers 4,000 and 900 cm^−1^. (a) Untreated SW620 cells (control). (b, c and d) SW620 cells treated with 50 μM 5-FU for 12, 24 and 48 h, respectively.

**Figure 5:**
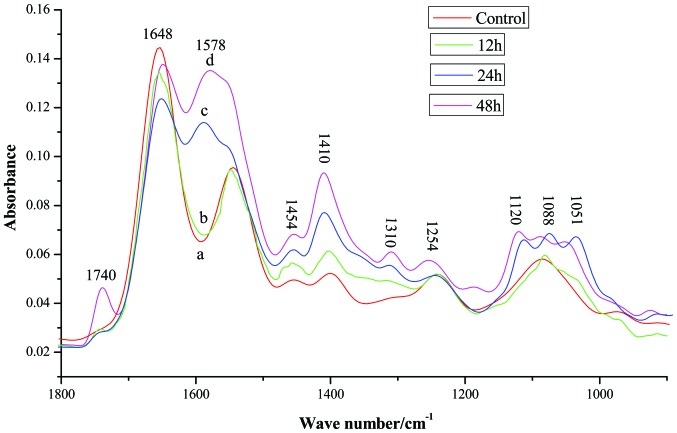
Fourier transform infrared spectra in the 1,800 and 900 cm^−1^ wave number region in the (a) control SW620 cells and (b-d) in the SW620 cells treated with 5-fluorouracil for (b) 12 h, (c) 24 h and (d) 48 h.

**Figure 6:**
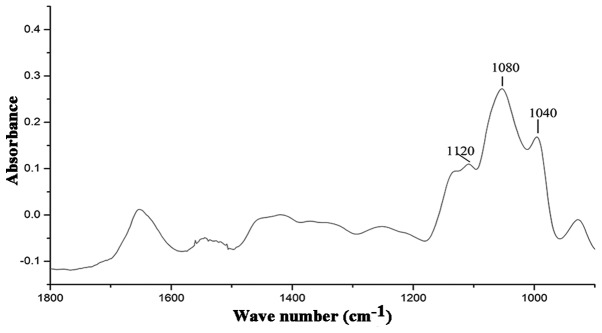
Fourier transform infrared spectra of the SW620 cell nuclei in the 1,800-900 cm^−1^ wave number region.

**Figure 7:**
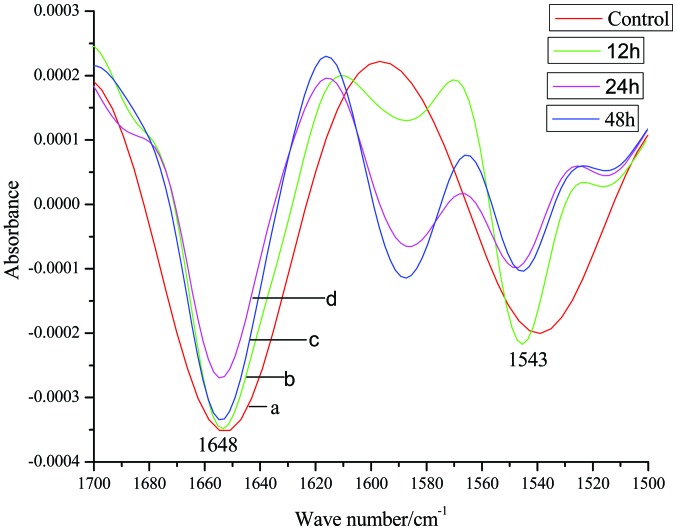
Derivative spectra of the 1,700-1,400 cm^−1^ region. (a) Control SW620 cells and (b–d) in the SW620 cells treated with 5-fluorouracil for (b) 12 h, (c) 24 h and (d) 48 h.

The absorbance spectra of the SW620 cells from the control group and the cells treated with 5-FU for 12, 24 and 48 h are shown in Fig. 4. The appearance of two new bands occurred at 24 h (Fig. 4C) at 1,120 cm^−1^ and 1,080 cm^−1^, respectively. Relevant absorbance bands in the FTIR spectra are shown in Fig. 5, between the fingerprint region of 1,800 and 900 cm^−1^. The new bands, which appeared at 24 h, were further examined by extraction of the nuclei of the SW620 cells to confirm whether 1,120 cm^−1^, representing threonine (Thr) and serine (Ser) and 1,080 cm^−1^ (nucleic acid molecule polysaccharide) were present in the nuclei (Fig. 6). The band assignments are shown in Table I. The second derivative spectra of 1,700-1,400 cm^−1^ in Fig. 7 were used to investigate amide I and amide II and the FTIR results were analyzed between the 4,000 and 900 cm^−1^ spectral regions.

### Band changes are associated with lipids

**Figure 8:**
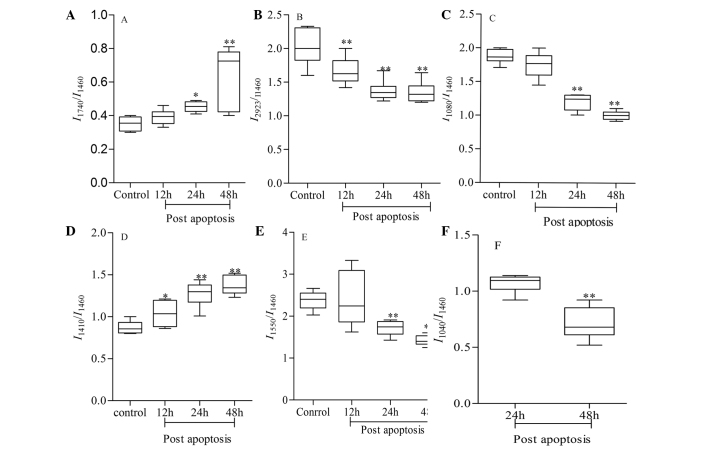
Statistical analysis of Fourier transform infrared relative intensity ratios (/*I*_1460_) of the control SW620 cells and the cells treated with 5-fluorouracil for 12, 24 and 48 h using one-way analysis of variance. (A) *I*_1740_/*I*_1460_, (B) *I*_2923_/*I*_1460_, (C) *I*_1080_/*I*_1640_, (D) *I*_1410_/*I*_1640_, (E) *I*_1550_/*I*_1640_ and (F) *I*_1040_/*I*_1640_. Boxes and error bars represent median, interquartile range and range, respectively.^*^P<0.05, compared with the control; ^**^P<0.001, compared with 12 and 24 h.

The changes in the relative intensity ratios of the main functional groups are shown in Fig. 8. The bands associated with cell lipids occurred mainly at 2,925, 2,852 and 1,740 cm^−1^. The 2,925 and 2,852 cm^−1^ spectral features were absorption bands of asymmetric and symmetric C-H stretching vibrations of CH_2_ and CH_3_ methylene groups, which are contained in fatty acids in cellular membranes (28). The band at 1,740 cm^−1^ is a C=O stretching vibration. Changes in these bands can reflect lipid changes in tissues and cells (29). Late apoptosis (48 h) was associated with large lipid-associated peaks (1,743 cm^−1^), the relative intensity ratios *I*_1740_/*I*_1460_ were significantly increased after 48 h compared with those of the control, 12 and 24 h groups, with an increase between 0.34±0.03 and 0.77±0.04 (P<0.001; Fig. 8A), whereas the relative intensity ratio *I*_2923_/*1*_1460_ was significantly decreased after 48 h compared with those of the control and the 12 and 24 h groups, which may indicate that the degree of apoptotic cell methylation decreased (Fig. 8B).

### Band changes are associated with nucleic acids

**Figure 9:**
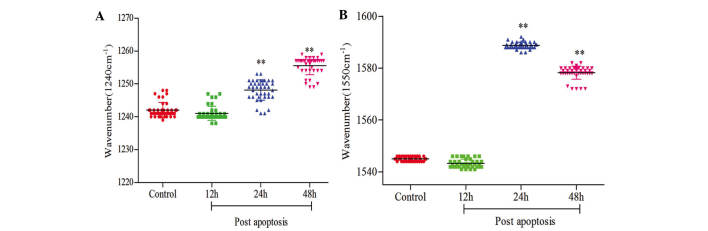
(A and B) Fourier transform infrared spectrum peak position comparisons between the control cells and the cells treated with 5-fluorouracil for 12, 24 and 48 h using one-way analysis of variance. ^**^P<0.001 compared with the control.

The two major bands in the regions of 1,080 and 1,240 cm^−1^ are mainly due to the symmetric and asymmetric stretching modes of the phosphodiester groups (30,31). These two bands are associated with the nucleic acid content of a cell (32). The ratio of intensities at the absorption *I*_1080_/*I*_1640_ began to decrease at 12 h and the ratio was significantly lower at 48 h (P<0.001; Fig. 8C), with late apoptotic DNA degraded into small fragments. The morphological results of the apoptotic bodies from the phagocytosis of DNA fragments morphological results coincided with this. The band at the 1,080 cm^−1^ absorption peak shifted to lower wave numbers at 12 and 24 h; however, the shift to a higher wavenumber at 48 h may have been due to enhancement of hydrogen bonds in the late apoptotic stage (Fig. 9A). Further investigation is required to investigate the underlying causes.

### Band changes are associated with amino acids

The vibrational bands at 1,120 cm^−1^ appeared at 24 h (early apoptosis) and increased at 48 h (late apoptosis) compared with those in the other groups. The 1,120 cm^−1^ absorption band was mainly due to ser, thr and tyrosine C-O (H) stretching vibration (Figs. 4C and D and 5). FACS can detect apoptotic cells as Annexin V has a high affinity for phosphatidylserine. Fig. 3E shows Annexin V binding at the different stages of apoptosis (24 and 48 h). Annexin V binding to phosphatidylserine increased over time, indicating that ser increased the exposure of phosphatidylserine on the outer lipid layer, consistent with the FTIR spectra results.

The band at the 1,410 cm^−1^ peak, which represents C-H stretching-associated amino acid residues, shifted to higher wave numbers compared with those in the other groups (Fig. 8D).

### Band changes are associated with amide I and II

In all spectra, major peaks were observed for absorptions in the amide I and amide II regions at 1,640 and 1,550 cm^−1^, respectively. The vibrational band at 1,640 cm^−1^ was primarily characterized by the α-helix secondary structure of the proteins (33) and the absorption bands at 1,550 cm^−1^ were attributed to the β-sheet secondary structure of proteins (34). As shown in Fig. 7, the spectral intensity at 1,640 and 1,550 cm^−1^ decreased in the second order derivative spectra of the region of 1,700-1,400 cm^−1^ at early and late stages of apoptosis. This decrease in intensity indicated that the α-helix and β-sheet contents of the apoptotic cells decreased. The relative peak intensity ratio *I*_1543_/*I*_1460_ increased significantly, indicating that the hydrogen bond constraint was reduced (Fig. 8E) and the band at 1,550 cm^−1^ shifted to higher wave numbers at 24 and 48 h (Fig. 9B).

### Band changes are associated with polysaccharides

The absorption band at 1,040 cm^−1^ was mainly due to the polysaccharide C-O stretching vibration arising in early apoptosis (35); however, the peak intensity decreased in late apoptosis (Fig. 8F), suggesting that polysaccharides decreased in late apoptotic cells.

## Discussion

The present study demonstrated that the FTIR-MSP spectral pattern of cells of the SW620 human colon cancer cell line reflects their apoptotic stage. On comparison of the spectral analysis with the FACS data, the most obvious differences were observed between 24 h (early apoptosis) and 48 h (late apoptosis). Flow cytometric data revealed that the number of apoptotic cells increased significantly after 24 h and 48 h compared with the cells in the control group and at 12 h.

Apoptosis was characterized by changes in four IR biomarkers: Increased lipid content and absorbance of ser and thr at 1,740 cm^−1^ and decreased DNA absorbance, decreased α-helix and β-sheet secondary structures of total cellular proteins within the apoptotic cells, the appearance of new vibrational bands at 1,120 and 1,040 cm^−1^ at 24 h with a decrease in the band at 1,040 cm^−1^, associated with polysaccharide absorbance at 48 h and an increase in amino acid residues.

Despite several tools to monitor and assess the curative effect of chemotherapy on cancer, colon cancer remains the most frequent cause of mortality in cancer patients. Thus, the investigation of techniques to monitor colon cancer chemotherapy is of clinical importance.

Compared with established techniques, the IR method is relatively inexpensive, fast and can be automated with no reagents. In conclusion, the present study demonstrated that FTIR spectroscopy can distinguish cell apoptosis based on changes in DNA conformation and protein secondary structure, particularly alteration in amino acid residues. This may provide a promising novel tool to monitor cell death in cancer chemotherapy clinically.

## Figures and Tables

**Table I:** Band assignments of major absorptions in the Fourier transform infrared spectra of the SW620 cells in the 4,000-900 cm^−1^ region.

Peak number	Frequency (cm^−1^)	Assignments
1	3,290	ν_O-H,_ ν_N-H_ (water, protein)
2	2,925	C-H stretching bands ν_as_ CH_2_ (lipid)
3	2,852	ν_s_ CH_2_ of lipids
4	1,740	C=O stretching bands (lipids)
5	1,640	Amide I (of proteins in α-helix conformation) and water
6	1,540–1,580	Amide II (an N-H bending vibration coupled to C-N stretching)
7	1,454	CH_3_ bending vibration (lipids and proteins)
8	1,410	δC-H, δC-O-H, amino acid residues
9	1,310–1,320	Amide III band components of proteins
10	1,240	Asymmetric PO_2_^−^ stretching in RNA
11	1,121	C-O(H) stretching bands, threonine and tyrosine
12	1,080–1,085	C-O-C stretching (nucleic acids and phospholipids) indicates a degree of oxidative damage to DNA
13	1,040	C-O stretching bands, nucleic acid molecule polysaccharide
